# How do family doctors respond to reduced waiting times for cancer diagnosis in secondary care?

**DOI:** 10.1007/s10198-023-01626-2

**Published:** 2023-10-03

**Authors:** Helen Hayes, Rachel Meacock, Jonathan Stokes, Matt Sutton

**Affiliations:** 1 Office of Health Economics (OHE), London, UK; 2 Health Organisation, Policy and Economics (HOPE), Centre for Primary Care & Health Services Research, School of Health Sciences, The University of Manchester, Manchester, UK; 3 MRC/CSO Social & Public Health Sciences Unit, School of Health and Wellbeing, University of Glasgow, Glasgow, UK; 4 Melbourne Institute of Applied Economic and Social Research, Faculty of Business and Economics, The University of Melbourne, Parkville, VIC Australia

**Keywords:** Waiting times, Demand elasticity, Early detection of cancer, Referrals, Family doctors

## Abstract

Reducing waiting times is a priority in public health systems. Efforts of healthcare providers to shorten waiting times could be negated if they simultaneously induce substantial increases in demand. However, separating out the effects of changes in supply and demand on waiting times requires an exogenous change in one element. We examine the impact of a pilot programme in some English hospitals to shorten waiting times for urgent diagnosis of suspected cancer on family doctors’ referrals. We examine referrals from 6,666 family doctor partnerships to 145 hospitals between 1st April 2012 and 31st March 2019. Five hospitals piloted shorter waiting times initiatives in 2017. Using continuous difference-in-differences regression, we exploit the pilot as a ‘supply shifter’ to estimate the effect of waiting times on referral volumes for two suspected cancer types: bowel and lung. The proportion of referred patients breaching two-week waiting times targets for suspected bowel cancer fell by 3.9 percentage points in pilot hospitals in response to the policy, from a baseline of 4.8%. Family doctors exposed to the pilot increased their referrals (demand) by 10.8%. However, the pilot was not successful for lung cancer, with some evidence that waiting times increased, and a corresponding reduction in referrals of −10.5%. Family doctor referrals for suspected cancer are responsive at the margin to waiting times. Healthcare providers may struggle to achieve long-term reductions in waiting times if supply-side improvements are offset by increases in demand.

**Supplementary Information:**

The online version contains supplementary material available at 10.1007/s10198-023-01626-2.

## Introduction

Waiting times are a common feature of publicly funded health systems, where demand exceeds capacity and must be limited by means other than consumer price [1]. Long waiting times are associated with patient dissatisfaction, utility loss during the waiting period, and potentially a reduction in health gains [2, 3]. Policies to reduce waiting times are therefore frequently implemented. These policies most often target supply-side responses with the aim of increasing capacity and/or efficiency. However, the demand response that may be induced as a result should be considered when assessing overall intervention impact [4].

Waiting times can affect both supply and demand. On the supply side, longer waiting times may encourage altruistic providers to increase activity to avoid patients experiencing the costs of waiting. In countries where waiting times are used as performance indicators, higher waiting times may also encourage increased activity through punishment for missed waiting times targets [2, 5]. On the demand side, patients may be dissuaded from treatment altogether if the wait is too long, or may opt to pay privately [2]. Most studies estimate the waiting-time elasticity of demand to be negative and generally low, meaning reductions in waiting times are offset by only small increases in demand [4, 6–8]. Elasticities of supply with respect to waiting times are generally found to be positive, but estimates vary more depending on context [9].

In many countries, general practitioners (GPs) act as gatekeepers for specialist hospital services [10]. GP demand for specialist hospital appointments for their patients is based on a complex set of decisions and trade-offs: GP factors (for example risk tolerance, experience and training); patient factors (symptom severity, anxiety, desire for referral); and structural factors (appointment availability, distance, regulation) [11]. GPs report a willingness to change their referral behaviour for non-urgent procedures in response to shorter waiting times [12, 13]. [14] found GP demand for outpatient hospital appointments for their patients was negatively affected by waiting times. Whilst an increase in supply reduced waiting times in the short-term, the demand response to these shorter waiting times meant system-level waiting times returned to original levels in the longer term.

These studies highlight the complexity of tackling waiting times in healthcare, because reforms aiming to reduce waiting times may not be successful in the long term if demand is sufficiently elastic. However, the existing literature has focused on GP referrals for elective or non-urgent care. GPs may behave differently when dealing with more urgent issues, such as suspected cancer. This has not yet been explored empirically.

There is increasing focus on early detection and diagnosis of cancer, due to the reduced cost of treatment and higher chance of survival [15, 16]. England performs poorly in international comparisons of cancer survival rates, which has been partly attributed to later presentation to services [17]. Speeding up time to diagnosis has therefore been a major policy goal over recent years.

In 2017, five hospitals in England became pilot locations for a new stricter maximum waiting time standard covering the time to a confirmed diagnosis, or ruling out, of cancer. The aim of this paper is to examine the GP demand response to this reduction in waiting times. We first examine whether the standard led to reduced waiting times in the pilot locations. We then evaluate the demand response from GPs in terms of the volume of referrals generated.

## Background

### The role of general practitioners in the cancer referral pathway in England

For the patient, most healthcare is free at the point of use and funded through general taxation. Individuals in England register with a single GP practice, and access appointments for routine and urgent primary care through this practice. Patients can directly use hospital services in an emergency but must obtain a referral from a GP for non-emergency care.

GPs are paid by capitation. GP quality is incentivised through a published survey of access, a considerable amount of quality reporting, and patients are free to register with a different practice at any time [18].

A GP who suspects cancer can request an urgent referral for the patient to a specialist hospital doctor. An existing waiting times target, introduced in 2000, states this appointment should take place within two weeks of referral. This referral pathway is unique to cancer.

### Cancer waiting times in England

**Fig. 1:**
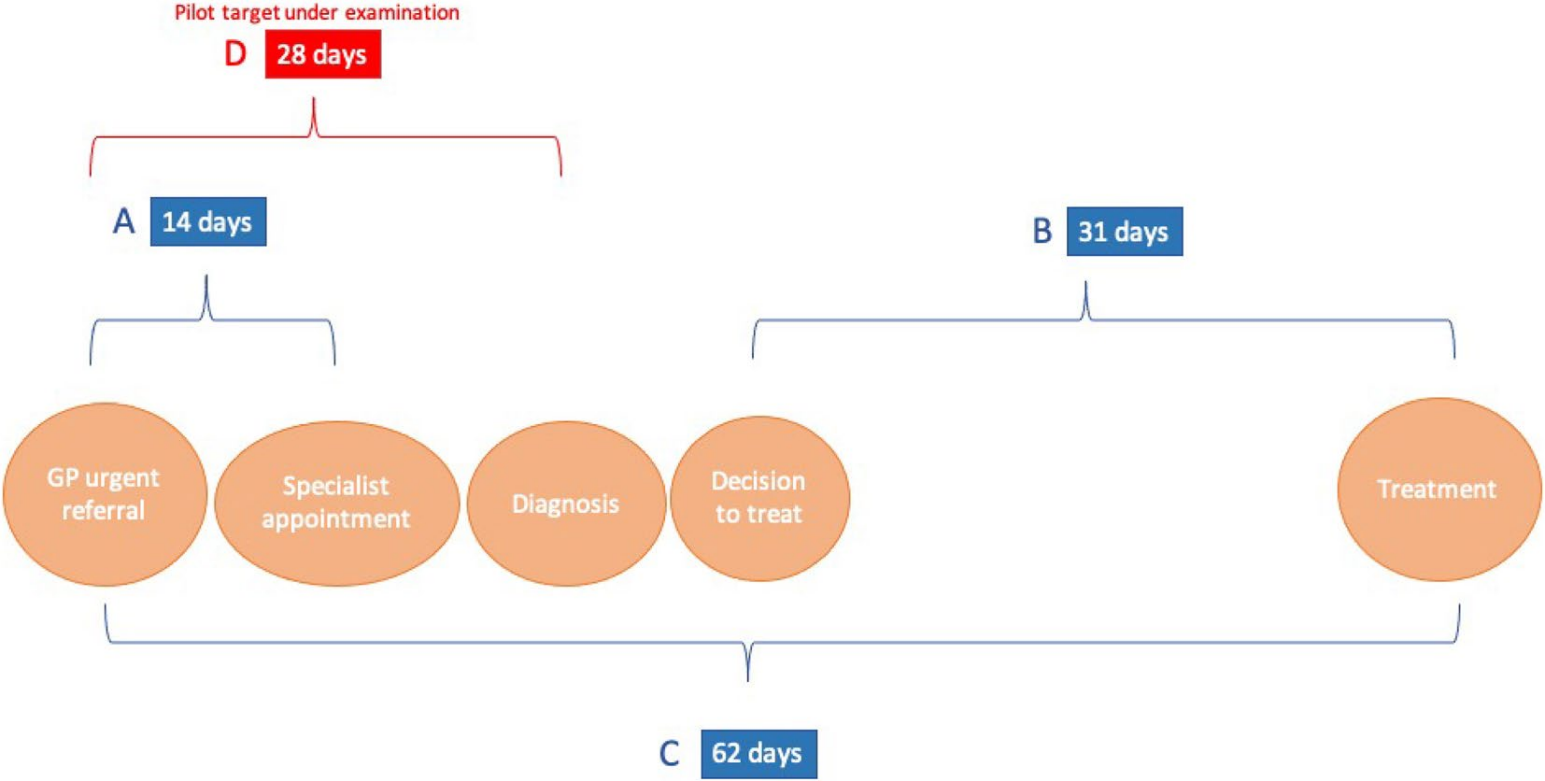
Cancer waiting time targets in England

Many health systems employ maximum waiting times policies, stating patients should not wait longer than a certain period before receiving care [2]. In England, hospitals are required to meet an operational standard based on the percentage of patients who meet a maximum waiting times target, which are enforced primarily through public reporting of performance. There were eight existing cancer waiting times targets in place during the period of our analysis. These targets fall into the following three groups (see Fig. 1) [19]:

A. Two-week wait from GP urgent referral to the first specialist consultation
B. 31-day wait from specialist decision to treat to first treatment
C. 62-day wait from GP urgent referral to first treatment

In 2017, 5 of the 152 acute hospital Trusts in England became pilot locations for an additional waiting time target. This additional target stipulated patients would receive a definitive diagnosis or ruling out of cancer within 28 days of urgent GP referral under the ‘28-day faster diagnosis standard’.

Figure 1 shows where the new target (target D) fits on the timeline from referral to treatment.

There has been a general trend of worsening adherence to cancer waiting times standards over time in England [19]. Prior to the introduction of the pilot 28-day faster diagnosis standard, performance against the waiting times targets covering time from GP referral to first specialist consultation (A) and time from decision to treat to first treatment (B) was very high [19]. However, performance against the target covering the full treatment pathway, time from GP referral to first treatment (C), was poor. The 28-day faster diagnosis standard was intended to address this failing, introducing a specific standard to target the element of the care pathway covering time to diagnosis, where delays were previously occurring [19].

If met, the previous targets (A, B, C) placed implicit bounds on time limits along the care pathway. Assuming the decision to treat happens immediately after diagnosis, and providers take the full length of time for each target, target C combined with target B leaves a maximum of 31 days between urgent GP referral and diagnosis if all three targets were to be just met. This would mean the new target (D) encouraged a reduction of three days in time to diagnosis, translating to a 10% reduction in the time to diagnosis compared with the previous pathway. Furthermore, three days is a conservative estimate of how much the new standard could reduce time to diagnosis. If the time between diagnosis and treatment can happen quickly, and the majority of the 62 day period was previously being taken up by delays between first specialist appointment and diagnosis, then the implementation of the new standard could reduce time to diagnosis by up to a month.

Target C was increasingly not being met in practice, meaning patients in many cases waiting longer than 31 days to receive a diagnosis. At the start of 2017, target C was being met for less than 80% of patients [19]. Therefore, the introduction of this new faster diagnosis standard would likely encourage a reduction in the time to diagnosis greater than 3 days.

### The reform under examination

#### The pilot sites and cancer pathways covered

The aim of the 28-day faster diagnosis standard pilot was to test, evaluate, and provide feedback on rules and definitions required in measuring adherence to the new target, and to provide information on additional capacity needed [17]. The pilot hospital Trusts were Leeds, East Lancashire, Kingston, Royal Bournemouth and Christchurch, and Ipswich. Whilst the existing national waiting times targets apply to all cancer types (see Fig. 1), the practicalities of delivering care differ between cancer types in terms of symptoms leading to referral, specialists seen, and diagnostic and treatment resources used. During the pilot, each hospital focused on piloting the 28-day faster diagnosis standard for at least two of six types of cancer (see Supplementary Table 1).

The programme encouraged Trusts to develop their own ways to reduce waiting times by making changes such as redesigning and streamlining care processes and pathways, and altering workforce inputs. We hypothesise the response to the pilot only applies to patients with suspected cancer types for which the waiting times target was being piloted, as waiting times targets for other cancer types remained unchanged during the period. Data on waiting times and GP referrals are only published by specific cancer type for two of the types of cancers covered by the pilots: bowel and lung cancer. We, therefore, focus our respective analyses on the three pilot Trusts (Ipswich, Kingston, and Royal Bournemouth and Christchurch) that focused on bowel cancers and the two Trusts (East Lancashire and Royal Bournemouth and Christchurch) that focused on lung cancer [20].

Bowel and lung cancer are common and deadly and benefit from early diagnosis. Bowel cancer is the fourth most common cancer in the UK and the second most common cause of cancer death. Lung cancer is the third most common cancer in the UK, but the most common cause of cancer death 2018. Both types of cancer have greatly improved survival rates when diagnosed at the earliest stage compared to the latest stage [21, 22].

Early detection for bowel cancer can be difficult given the frequent lack of discriminating presenting symptoms [23], and for lung cancer as there are usually no symptoms at the early stages [24]. There is scope for improvement of early diagnosis, which likely explains why they were included in the pilots. Compared to bowel cancers, which generally grow slowly [25], lung cancer patients tend to present with symptoms at a later and more urgent stage.

#### Waiting times incentives for hospital trusts

Incentives for hospital Trusts in the UK to adhere to waiting times standards are a combination of reputational and financial. Providers can face financial penalties for failing to meet national waiting times targets [26], but there were no financial penalties applied to the 28-day target during the pilot period. Incentives for adherence to the pilot programme were therefore primarily reputational.

There are strong reputational incentives for hospital Trusts in the UK to meet their waiting times targets. Hospital performance is published monthly and closely monitored by national policymakers and regulators [27]. Senior NHS managers are frequently threatened with job losses as a result of failing to meet waiting times targets [28, 29] Participating in the pilot would have likely come with additional reputational pressures, as the pilot was closely monitored by NHS England. Reputational incentives have previously been shown to have significant impacts on performance of health care organisations in the UK [28, 30, 31].

#### Timelines and characterisation of the supply shifter

Pilot sites were chosen and rules developed during 2016/17 (1st April 2016–31st March 2017). Exploration of the 28-day faster diagnosis standard on service delivery began in 2017/18. The financial year 2017/18 is taken as the first year of the reform [32]. We study its impact over the period 2017/18 and 2018/19, before it was rolled out nationally in quarter three of 2021/22 [33].

When estimating demand or supply responsiveness to waiting times, generally only a measure of waiting times or activity can be directly observed, and not whether different activity levels correspond to supply or demand variations. Estimating the effect of waiting times on demand therefore requires the use of a variable which impacts waiting times but not underlying demand [2]. In the pilot hospital sites, steps were taken to improve services and increase capacity to enable shorter waiting times for diagnostic services. We expect waiting times to improve in pilot sites relative to the rest of England. We use the pilot as a ‘supply-shifter’ to estimate the effect of waiting times on demand.

### Potential mechanisms driving GP demand response to the pilot waiting time target

We investigate whether GPs’ referral behaviours respond to the reduction in waiting times for diagnosis on the suspected cancer treatment pathway. We hypothesise that, regardless of the pilot waiting times target, GPs would always refer patients whom they suspect to have cancer. We, therefore, do not expect to see a change in GP referral behaviour in response to shorter waiting times for these patients. Instead, we hypothesise that shorter waiting times may influence GPs’ decisions and threshold for referral at the margin, amongst patients for whom the GP believes that cancer is less likely. A priori, we therefore hypothesise that GPs may respond to reduced waiting times on the cancer referral pathway in two possible ways. These responses are detailed in Table 1.

**Table 1:** Potential general practitioner demand responses to a reduction in waiting times for suspected cancer referrals

Volume of referrals (demand response)	Mechanism driving referral volumes to change
⇑	GP knows the diagnostic process is shorter and is therefore more inclined to refer a patient in order to rule out cancer quickly for patients they do not suspect have cancer
⇓	Knowledge of reduced waiting times acts as a safety net, giving more scope for GPs to explore other avenues of diagnosis before referral in the knowledge that the diagnostic process will be faster if these investigations do not turn out to be definitive

Amongst patients who GPs do not strongly suspect cancer, or where the diagnosis is less obvious, a reduction in the time to diagnosis receipt may induce GPs to refer more patients onto the cancer pathway in the hope of ruling out cancer to reduce the period of uncertainty for the patient. Models based on the demand response to waiting times for non-urgent or elective surgery predict an increase in demand in response to shorter waiting times [12, 13]. If a GP knows that the diagnostic process is shorter, they may be more inclined to refer a patient in order to rule out cancer.

Even small delays in a cancer diagnosis can have important implications for patient outcomes. The intensity of management of cancer waiting times, including standards and targets specifically placed on this part of the pathway, demonstrates the importance given to reducing delays in this early detection phase. Cancer diagnostic delay has been shown to have lasting effects on patients’ psychological wellbeing, levels of distress and quality of life [34], and so any reduction in this waiting time is valued by patients. As GPs display a level of altruism towards their patients, reducing the length of this psychological distress is likely to also be valued by GPs, with some agents, therefore, likely to respond on the margin.

Alternatively, the knowledge that waiting times in specialist care have been reduced may act as a safety net for GPs who do not suspect cancer, allowing GPs more scope to explore other alternatives first for patients they do not suspect have cancer. If some of these cases are subsequently resolved through GP investigation, this would result in a reduction in the volume of patients referred for suspected cancer. In 2016, the National Cancer Experience Survey reported that 26% of responding patients subsequently diagnosed with cancer saw their GP three or more times before being referred to specialised hospital services [35]. Signs of cancer are not always clear and distinctive from other less serious conditions (such as weight loss and fatigue). With over 200 types of cancer, many of which are associated with vague symptoms, GPs must find the balance between referring all potential cases and inducing unnecessary anxiety and discomfort through further testing [36]. This decision takes place within a limited appointment time (usually 10 min in England). GPs’ incentives for referral are not only influenced by GP and patient factors, but structural factors too. GPs in the UK are aware of the rising pressures facing NHS hospitals, and, during the period of our analysis, many had been offered financial incentives to reduce the number of referrals to hospitals, including urgent referrals for suspected cancer [37, 38]. In summary, the incentives for referral are not straightforward, and responses could plausibly go in either direction.

If a GP demand response does occur in either direction, then this must include a change in the total volume of referrals for patients which GPs do not strongly suspect of cancer, rather than purely a diversion effect of patients who would be referred anyway to a different hospital, now being referred to one of the pilot hospitals. Diverting patients away from non-pilot and towards pilot Trusts would still be a response to the waiting times change, but would not represent a change in overall GP demand as we outline in Table 1.

Given the characteristics of these cancer types, we hypothesise that the option of first exploring other alternatives may be more viable for bowel cancer as opposed to lung cancer (due to, for example, the later presentation of lung cancer symptoms and the relatively indiscriminate nature of bowel cancer symptoms combined with, on average, slower growth).

#### Receipt of information regarding waiting times by GPs

Information on the pilot/waiting times would have reached GPs in at least three ways. Firstly, the pilot was publicised through press releases and NHS England blogs (Palmer, 2017), and GP practices were sent emails about these updates. GPs would have, therefore, likely been aware that the pilot was happening at their local hospital. Secondly, hospital performance against the national waiting times targets is published monthly [27], with a three month delay. Thirdly, GPs will have been aware of how long their own patients were waiting. The average GP practice makes over 300 suspected cancer referrals a year, approximately 65 per FTE GP [39]. GPs, therefore, repeatedly observe waiting times for their own patients throughout the year, giving them direct personal experience of whether these are changing over time.

## Methods

We use two separate datasets. Our analysis of waiting times is carried out at the hospital Trust level. We analyse the GP demand response at the GP practice level. We consider bowel cancer and lung cancer separately throughout.

### Waiting times

#### Data

##### Sample and time periods examined

Our sample consists of 145 acute hospital Trusts in England. Of the 152 hospital Trusts open when the pilot was introduced, we exclude Trusts which did not treat any suspected lung or bowel cancer patients from our analysis. The panel is unbalanced as some hospital Trusts opened, closed, or merged with another Trust during the analysis period.

For the lung cancer analysis, we use annual hospital Trust data between the financial years 2012/13 and 2017/18. This covers 5 years before the reform and 2 years after, stopping before the standard was applied nationally. The dataset is constructed from a number of sources from NHS England, NHS Digital and GOV UK that are linked by the provider code [40–43]. Supplementary Table 2 includes a full description of the sources of each of the variables described in Sect. "Variables".

In 2018/19, one of the three bowel cancer pilot sites, Ipswich Hospital NHS Trust, merged with another hospital Trust which was not involved in the pilot (Colchester Hospital University Foundation Trust). Therefore, in our main analysis of the bowel cancer pilot, we include the two hospitals separately but stop our analysis before they merged, analysing the impact of the reform in its first year only.

##### Variables

*Outcome variables* Data on exact waiting times from urgent referral to diagnosis are not published at the hospital Trust level so data on performance against the 28-day faster diagnosis standard is not available during the pilot period. Instead, we use a related measure of waiting times performance; the number of breaches of the existing waiting times target (A) 14 days from GP urgent referral to first specialist appointment (Target A, Fig. 1). This two-week wait rule covers the first part of the pathway and is embedded within the new pilot standard which also encompasses the following step to diagnosis. We define our waiting times outcome as the percentage of patients referred for suspected cancer for whom the two-week wait target is breached. We will refer to this as “waiting time breaches, as a % of total suspected cancer appointments”. We use waiting time breaches for bowel and lung cancers in each respective analysis as the best available proxy for longer waiting times.

*Covariates* We use several provider-level covariates. These are not available specifically for cancer services, but instead reflect time-varying measures of general hospital Trust-level capacity and quality. We include a number of covariates that may be correlated with the pilot and may influence waiting times to mitigate omitted variable bias. Furthermore, inclusion of appropriate covariates in difference-in-differences (DiD), our main empirical approach, can reduce residual variance and improve the precision of the DiD estimate (Cunningham, 2020). We include measures of activity and staffing which control for general hospital supply changes (full-time equivalent (FTE) all staff, total number of beds available, and proportion of beds occupied. For quality indicators we use the waiting list size for elective operations as a measure of access to care, and the mean length of stay for all spells of continuous admitted patient care as a measure of efficiency.

##### Treatment definition

The waiting times analysis is conducted at the hospital Trust level, with the treatment group defined as the pilot Trusts. For bowel cancer, the treatment group consists of the three hospital Trusts which focused on bowel cancer for their 28-day faster diagnosis pilot. For lung cancer, the treatment group consists of the two hospital Trusts which focused on lung cancer. For each analysis, the control group consists of all other hospital Trusts in England.

#### Empirical strategy

We use DiD methods to evaluate the effect of the pilot on waiting times, estimated with the following two-way fixed effects ordinary least squares (OLS) model:

1 $${W}_{ht}={\beta }_{0}+ {{\beta }_{1}FDS}_{h}*POS{T}_{t}+{{\varvec{\beta}}}_{2}{{\varvec{X}}}_{{\varvec{h}}{\varvec{t}}}+{\alpha }_{h}+{\tau }_{t}+{\varepsilon }_{ht},$$

where ${W}_{ht}$ represents the rate of waiting time breaches for hospital Trust $h$ in year $t$. ${FDS}_{h}$ is a treatment dummy equal to 1 if a hospital Trust is one of the pilot sites and 0 otherwise. $POS{T}_{t}$ is a dummy variable equal to 1 for observations in 2017/18 and 2018/19, and 0 otherwise. ${{\varvec{X}}}_{{\varvec{p}}{\varvec{t}}}$ are the Trust-level, time-varying covariates. ${\alpha }_{h}$ denotes a hospital Trust fixed effect and ${\tau }_{t}$ is a time-fixed effect which controls for time-varying factors which are fixed across hospitals.

#### Parallel trends and the implication of pre-trends for the estimated pilot effect

The parallel trends assumption states that the trend in the outcome of interest would have been the same for the comparison group as the treatment group in the absence of the intervention. It is a key assumption of DiD analysis. Observation of trends in the pre-period are generally used as a guide as to whether it is likely to be met. We carry out F-tests of the treatment and time trend interaction in the pre-period, which indicates whether trends are diverging systematically before the pilot was introduced.

Recent DiD literature has questioned the simplicity of the pre-trends test in evaluating the validity of the parallel trends assumption, and has explored ways of adjusting for the extrapolation of pre-trends [44, 45]. In our analysis, we assume the difference in trends, which we estimate using the pre-period data, persists into the post-period, and we discuss the implication of these differences in relation to the estimated treatment effect. This approach moves beyond reliance on arbitrary cut-offs for statistical significance, and instead interprets the direction and magnitude of any divergence in pre-trends in relation to the estimated treatment effect. The intention is to make transparent the assumptions needed in order to draw conclusions around treatment effectiveness.

### GP response

#### Data

##### Sample and time periods examined

We use annual practice level data on 6,666 general practices in England observed with full covariate information in at least 1 year between the financial years 2012/13 and 2018/19. Data are taken from Public Health Profiles provided by Public Health England [46]. The data used in Public Health Profiles are compiled from a variety of sources: NHS England, NHS Digital and the GP Patient Survey [40, 47, 48]. Supplementary Table 2 contains a full list of the sources of each variable for each dataset. The Public Health Profiles data includes practices with a list size of over 1,000 patients, participating in the Quality and Outcomes Framework (QOF) or for whom GP Patient Survey data is available from the GP Patient Survey. The panel is unbalanced as some practices opened and closed during the analysis period.

##### Variables

*Outcome variables* We examine volumes of urgent referrals for suspected bowel cancer and lung cancer as outcome measures of the GP demand response to the pilot.

We analyse the inverse hyperbolic sine transformations of the volumes to make the interpretation of results easier, approximate a normal distribution, and to reduce the effect of outliers [49]. Histograms of the outcome variables show more normality after transformation (see Supplementary Fig. 1).

*Covariates* We include a rich set of covariates which may be correlated with waiting time and GP referral activity: GP practice size (log of registered population); characteristics of the registered practice population’s age (proportion aged under 18, proportion aged 65 +), health (proportion with a long-standing condition) and employment status (proportion unemployed); patient-reported access (proportion reporting good experience of making an appointment, proportion satisfied with phone access); and clinical quality (QOF) points achieved across all domains as a proportion of all achievable points). The QOF measures primary care practice performance against a number of quality indicators [50]).

##### Treatment definition

When analysing practice-level referral rates, it is necessary to link practices to the hospital Trusts to which they make referrals to determine which practices were exposed to the hospital-level pilot intervention. Most general practices refer to more than one hospital Trust (see Supplementary Fig. 2 for the distribution of the shares of practice referrals to providers).

We would expect the pilot to induce a greater demand response from practices which refer mainly to the pilot sites than from GP practices which never or rarely refer to the pilot sites. We use the number of urgent two-week wait referrals for suspected cancer that GP practices made to each hospital Trust in 2016/17 (the year before the pilot took effect) to define exposure to treatment. These volumes were calculated using Hospital Episode Statistics (HES) data. From this data we create a ‘dose’ variable, equal to the proportion of a practice’s total urgent two-week wait referrals which were made to the relevant pilot locations in the year before the pilot was introduced. We do not have data on referrals by cancer type in HES. The dose variable is calculated as the proportion of a practice’s total suspected cancer referrals which were made to the three bowel cancer pilot sites for the bowel cancer analysis, and proportion of a practice’s total suspected cancer referrals which were made to the two lung cancer pilot sites for the lung cancer analysis. Given that we define the treatment dose based on referral patterns before the merger between Ipswich and Colchester NHS Trust, we ignore the post-pilot merger in the bowel cancer analysis treating the hospitals as two separate units when calculating the dose variable.

#### Empirical strategy

Using the measure of dose of exposure to treatment, we implement a continuous DiD design [51], as outlined in Eq. 2:

2 $${Y}_{pt}={\gamma }_{0}+ {{\gamma }_{1}DOSE}_{p}*POS{T}_{t}+{{\varvec{\gamma}}}_{3}{{\varvec{X}}}_{{\varvec{p}}{\varvec{t}}}+{\alpha }_{p}+{\tau }_{t}+{\varepsilon }_{pt},$$

where ${Y}_{pt}$ are the outcome variables for GP practice $p$ in year $t$. ${DOSE}_{pt}$ is the treatment dose variable outlined in 3.2.1.3. $POS{T}_{t}$ is a dummy variable equal to 1 for observations in 2017/18 and 2018/19, and 0 otherwise. ${{\varvec{X}}}_{{\varvec{p}}{\varvec{t}}}$ are the practice-level, time-varying covariates. ${\alpha }_{p}$ is a practice-fixed effect and ${\tau }_{t}$ denotes time-fixed effects. ${\varepsilon }_{pt}$ is the idiosyncratic error term. ${\gamma }_{1}$ is our coefficient of interest, capturing the effect of the pilot.

#### Parallel trends and the implication of pre-trends for the estimated pilot effect

As in 3.1.3, we carry out *F*-tests of the treatment dose and time trend interaction in the pre-period, which indicates whether trends are diverging systematically in the period before the pilot was introduced. We discuss not only the statistical significance of the pre-trends, but also the direction and magnitude of the effect in relation to the estimated treatment effect.

### Robustness checks

#### Alternative measure of waiting time

To alleviate concern that breaches of the 14-day waiting times target measure may not capture the effect of the pilot on the time between specialist appointment and receipt of a diagnosis, we carry out sensitivity analysis using an alternative measure of waiting time. We examine the impact of the pilot on breaches of the 62-day waiting times target (D in Fig. 1), which includes all of the 28-day period targeted by the pilot programme. We repeat the main analysis for waiting times, replacing the outcome with this new waiting times measure.

#### Alternative estimation in the absence of parallel trends

To address concerns that the parallel trends assumption may be violated, we repeat all of our analyses using the Lagged Dependent Variable (LDV) method as a means of estimating the causal effect of a policy in the absence of parallel trends. The LDV method adjusts for pre-treatment outcomes and covariates using a parametric regression model, and proxies the unobserved component using a fixed vector of pre-treatment outcomes. It assumes controlling for the pre-treatment effects is a sufficient proxy for all confounders [52].

For the waiting times analysis for lung cancer, there is concern that the parallel trends assumption is not met over the full 5-year pre-period we examine. We, therefore, repeat our main analysis over a shorter period of 3 years pre-pilot, where visual evidence suggests that the trends in treatment and control group appear more stable.

#### Treatment group definition

We carry out multiple checks to examine the robustness of our results to the definition of treatment. We conduct a sensitivity analysis of the bowel cancer waiting times analysis removing hospital Trusts involved in the merger (Ipswich and Colchester) from the sample entirely. This allows us to extend the post-treatment period to include 2018/19, examining the impact of the pilot for the remaining two pilot sites over 2 years post-reform. For the GP response analysis for bowel cancer, we carry out similar sensitivity analyses to address the merger. First, we conduct an analysis which excludes the merger year 2018/19, aligning with the waiting times sensitivity analyses. Secondly, we undertake another version removing all practices where the proportion of referrals to either Ipswich or Colchester Hospital Trust in 2016/17 (the year the treatment dose is defined) is greater than zero.

In our main analyses, the pilot sites not piloting the standard for bowel or lung cancer are included in the respective control groups. We examine the sensitivity of our results to excluding these remaining Trusts from the analysis altogether, i.e. excluding the two other pilot sites which were not specific to bowel cancer from our bowel cancer analyses, and excluding the three other pilot sites which were not specific to lung cancer from our lung cancer analyses.

Our main definition of GP’s exposure to treatment is based on GP referral behaviour in the year prior to reform, 2016/17. If GPs change which hospital Trusts they send their patients to over time, the dose measure of treatment exposure will be inaccurate. We test this using HES data from 2012/13 to 2017/18. We regress the proportion of referrals going to a pilot location on the covariates and year dummies using the year before the reform as the base category, to see if there are significant changes in the definition of the treated group over time compared to the year that they were defined.

Finally, we repeated the waiting times analysis, excluding all time-varying covariates.

## Results

### Waiting times

For bowel cancers, the existing two-week wait target was breached for patients with suspected bowel cancer in 4.78% of cases in pilot Trusts and 5.98% of cases in control Trusts in the period before the pilot was introduced (Table 2). After the pilot was introduced, waiting time breaches for suspected bowel cancers fell to 2.65% in the three bowel cancer pilot Trusts whilst rising to 7.54% in control Trusts. Both treatment and control Trusts experienced large increases in suspected bowel cancer referral volumes over this period.

**Table 2:** Average values of annual Trust-level data on the outcome variables and covariates by pre- and post-period, and treatment and control group

	Bowel cancer				Lung cancer			
	Pre-period		Post-period		Pre-period		Post-period	
	Control	Treatment	Control	Treatment	Control	Treatment	Control	Treatment
Waiting time breaches as a % of total suspected cancer patients appointments for respective cancer types	5.98	4.78	7.54	2.65	3.48	3.42	4.53	6.26
	(4.47)	(2.95)	(6.01)	(0.70)	(3.82)	(2.07)	(5.04)	(2.68)
Waiting time breaches for respective cancer types, *n*	111.13	70.43	200.45	53.67	14.18	16.20	20.57	31.25
	(118.99)	(54.58)	(244.97)	(14.29)	(17.38)	(9.26)	(25.04)	(12.26)
Total suspected cancer appointments for respective cancer types, *n*	1,735.04	1,419.50	2,427.25	2,025.67	401.98	483.00	471.37	517.50
	(802.96)	(368.89)	(1056.85)	(90.47)	(221.65)	(96.79)	(251.94)	(97.67)
FTE all staff, *n*	5,081.78	3,133.00	5,657.51	3,583.67	5,039.53	5,234.40	5,742.04	5,687.75
	(2,581.11)	(567.37)	(3,171.84)	(648.38)	(2,582.54)	(1,640.97)	(3,241.99)	(1,936.15)
Total beds, *n*	778.40	556.71	790.72	581.00	773.54	799.40	795.92	782.75
	(346.41)	(76.91)	(351.71)	(120.58)	(346.18)	(203.95)	(378.07)	(212.47)
Proportion of beds occupied	0.88	0.92	0.90	0.94	0.88	0.91	0.90	0.92
	(0.06)	(0.04)	(0.05)	(0.01)	(0.06)	(0.04)	(0.05)	(0.02)
Elective waiting list, *n*	38,171.16	31,831.00	40,453.69	30,821.67	37,941.01	44,990.90	41,272.22	44,791.25
	(18,662.37)	(10,035.90)	(20,891.05)	(8,985.54)	(18,660.17)	(1,936.35)	(21,643.82)	(5,710.51)
Mean length of stay, days	4.25	3.73	4.15	3.53	4.24	4.22	4.06	3.91
	(0.63)	(0.51)	(0.60)	(0.64)	(0.64)	(0.24)	(0.62)	(0.36)
Observations	678	14	130	3	682	10	259	4

*N* 145 acute Trusts. Standard deviations in parentheses below. For bowel and lung cancer respectively, waiting time breaches as a % of total suspected cancer appointments is bowel or lung cancer waiting times breaches as a % of total bowel or lung suspected cancer patients seen. Treatment and control are defined separately for bowel cancers and lung cancers. There is 1 year of post-period data for bowel cancer and two for lung cancer

For lung cancers, the existing two-week wait target was breached at similar rates in the pilot and control Trusts in the period before the pilot was introduced (3.42 and 3.48% of cases, respectively). After the pilot was introduced, breaches for suspected lung cancer rose to 6.26% in the two lung cancer pilot Trusts and 4.53% in control Trusts. Total referral volumes for suspected lung cancer also rose over the period for both groups.

Amongst the three hospital Trusts which piloted the faster diagnosis standard for bowel cancers, we estimate the pilot was associated with a 3.91 percentage point reduction in the number of breaches as a percentage of total suspected bowel cancer patient appointments (*p* < 0.01, Table 3). We do not detect a significant impact of the pilot on waiting times breaches for lung cancer.

**Table 3:** The effect of the pilot on waiting times for suspected bowel cancer and lung cancer

	Bowel cancer	Lung cancer
Pilot effect	−3.907***	1.695
	(1.344)	(1.310)
Total number of beds	1.850	−1.215
	(2.786)	(2.074)
Proportion of beds occupied	7.843	4.874
	(4.753)	(4.007)
FTE staff	1.527	0.966
	(2.564)	(2.193)
Elective waiting list size	−0.788	−0.277
	(2.084)	(1.581)
Length of stay	−0.132	−0.622
	(0.566)	(0.475)
Constant	−19.37	4.383
	(20.49)	(15.36)
Adjusted R2	0.0557	0.0339
N	825	955
Provider fixed effects	Yes	Yes
Year fixed effects	Yes	Yes

Total number of beds available and waiting lists are included as inverse hyperbolic sine transformations of the underlying counts. The bowel pilot includes three pilot Trusts, the lung pilot includes Two pilot trusts. Sample sizes vary between models as there is one year of post-period data for bowel cancer and two for lung cancer

Robust standard errors in parentheses. *p < 0.10, **p < 0.05, ***p < 0.01

#### Parallel trends and the implication of pre-trends for the estimated pilot effect

**Fig. 2:**
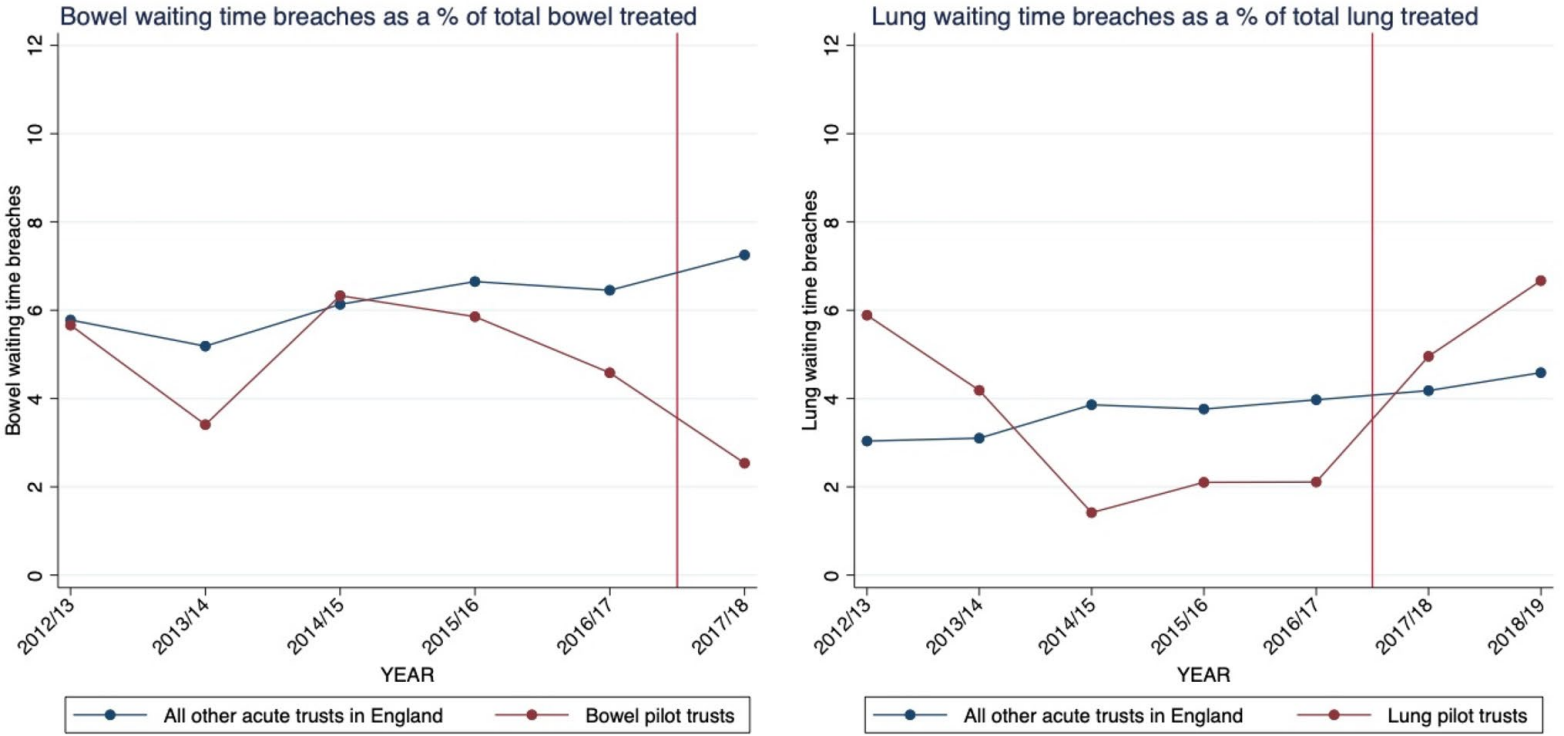
Adjusted plots over time for waiting time breaches in pilot Trusts vs all other Trusts in England. Graphs are adjusted for Trust-level covariates

For bowel cancer, there is some visual evidence that trends appear to be diverging slightly (Fig. 2), but the divergence in 2017/18 is much larger than what we would expect given the pre-period trends. The pre-trends tests in Table 4 show the estimated divergence in trends in the pre-period is −0.27 (*p* > 0.1). If we were to extrapolate this divergence into the post-period then our treatment estimate of 3.91 percentage points may be overestimated slightly. However, the divergence in parallel trends would not be enough to explain the entire magnitude of the effect.

**Table 4:** *F*-tests of parallel trends in waiting times breaches

	Bowel cancer	Lung cancer
Time trend*Treatment dummy	−0.269	−1.223**
	(0.433)	(0.533)
Time trend	0.291**	0.233***
	(0.121)	(0.0865)
Treatment dummy	540.3	2464.0**
	(871.9)	(1072.6)
*N*	692	692

Null hypothesis: parallel trends in waiting times breaches for treatment and control groups before the introduction of the pilot. Only observations in the pre-reform period (2012/13–2016/17) are included

Robust standard errors in parentheses. *p < 0.10, **p < 0.05, ***p < 0.01

For lung cancer, the visual evidence suggests the parallel trends assumption may not be met (Fig. 2). The estimated divergence of trends in the pre-period is −1.22 (*p* < 0.05) (Table 4), which is both statistically significant and sizable in magnitude compared to the estimated pilot effect (1.70, *p* > 0.1). However, as the direction of divergence in the pre-trends is opposite to that of the estimated treatment effect, if this divergence were to have persisted into the post-period this would mean our estimated pilot effect may be underestimated.

### GP referrals

For suspected bowel cancer, the rate of referral increased by 54% in the exposed practices in the post-period compared to before the pilot was introduced (3.95–6.07 per 1000 registered patient population) (Table 5). The rate of referral increased by 43% in control practices over the same period (4.12–5.91 per 1000 registered patient population).

**Table 5:** Average annual values of the GP practice-level outcome variables and covariates, by pre- and post-period, and treatment and control group

	Bowel cancer				Lung cancer			
	Pre-period		Post-period		Pre-period		Post-period	
	Control	Treatment	Control	Treatment	Control	Treatment	Control	Treatment
Number of two-week wait referrals per 1000 of the registered practice population	4.12	3.95	5.91	6.07	0.99	0.90	1.05	1.13
	(2.31)	(2.05)	(2.98)	(2.84)	(0.72)	(0.61)	(0.78)	(0.73)
Practice list size	7,727.40	8,681.15	8,472.24	9,501.60	7,784.06	7,674.15	8,523.34	8,771.61
	(4,507.07)	(4,504.19)	(5,307.76)	(5,291.67)	(4,516.36)	(4,373.09)	(5,308.37)	(5,431.85)
Proportion aged 65 + years	0.17	0.18	0.17	0.18	0.17	0.19	0.17	0.20
	(0.06)	(0.08)	(0.07)	(0.08)	(0.06)	(0.07)	(0.07)	(0.08)
Proportion aged under 18 years	0.21	0.20	0.21	0.19	0.21	0.21	0.21	0.20
	(0.04)	(0.04)	(0.04)	(0.04)	(0.04)	(0.04)	(0.04)	(0.05)
Total QOF points achieved (proportion of all achievable points)	0.96	0.96	0.97	0.97	0.96	0.97	0.97	0.98
	(0.06)	(0.06)	(0.06)	(0.06)	(0.06)	(0.05)	(0.06)	(0.05)
Working status—proportion unemployed	0.06	0.04	0.04	0.03	0.06	0.05	0.04	0.04
	(0.05)	(0.03)	(0.04)	(0.03)	(0.05)	(0.04)	(0.04)	(0.04)
Proportion reporting good overall experience of making appointment	0.75	0.77	0.70	0.73	0.75	0.77	0.70	0.72
	(0.13)	(0.12)	(0.15)	(0.13)	(0.13)	(0.13)	(0.15)	(0.14)
Proportion satisfied with phone access	0.76	0.78	0.72	0.77	0.76	0.79	0.72	0.77
	(0.17)	(0.16)	(0.19)	(0.17)	(0.17)	(0.16)	(0.19)	(0.16)
Proportion with a long-standing health condition	0.54	0.51	0.51	0.49	0.53	0.55	0.51	0.53
	(0.08)	(0.08)	(0.09)	(0.08)	(0.08)	(0.08)	(0.09)	(0.08)
Observations	30,605	1,816	12,441	748	31,466	955	12,802	387

*N* 6666 general practices. Treatment group is defined as practices for which the proportion of referrals to a pilot hospital in the year prior to reform > 0. Treatment and control are defined separately for lung cancer and bowel cancer. Standard deviations in parentheses below. Bowel urgent referrals and lung urgent referrals are weighted per 1000 of the population. All covariates with the exception of practice list size have a maximum value of 1

For suspected lung cancer referrals, there was a 26% increase in referrals from the treated practices over the period (0.90–1.13 per 1000 registered patient population) and a 6% increase from control practices (0.99–1.05 per 1000 patient population).

There was a statistically significant increase in referral volumes for suspected bowel cancer amongst practices exposed to the bowel cancer pilot (0.108, *p* < 0.01) (Table 6). This corresponds to an average relative increase in bowel cancer referral volumes of 10.8% for practices who referred all of their suspected cancer patients to a bowel pilot in the year before the pilot (i.e. treatment dose = 1), compared to practices who referred none of their suspected cancer patients to the three pilot Trusts. Our results, therefore, suggest that if bowel cancer waiting times breaches could be reduced at all Trusts in the country by the 3.91 percentage points detected in the pilots, an additional 35,731 patients would be referred per year.

**Table 6:** Continuous difference-in-differences estimates of the effect of the 28-day faster diagnosis standard pilot on referral volumes for suspected bowel and lung cancer

	Bowel cancer urgent referrals	Lung cancer urgent referrals
Continuous DiD	0.108***	−0.105**
	(0.0358)	(0.0460)
Registered practice population size	0.939***	0.841***
	(0.0309)	(0.0339)
Proportion aged 65 + years	−2.063***	−1.053**
	(0.366)	(0.453)
Proportion aged under 18 years	−0.855**	−0.371
	(0.390)	(0.453)
Total QOF points achieved (proportion of all achievable points)	0.0724	0.213***
	(0.0574)	(0.0785)
Working status – Proportion unemployed	−0.144**	−0.0423
	(0.0713)	(0.1000)
Proportion reporting good overall experience of making appointment	0.00371	0.00936
	(0.0400)	(0.0537)
Proportion with a long-standing health condition	−0.107***	−0.107**
	(0.0368)	(0.0504)
Proportion satisfied with phone access	0.0261	0.0435
	(0.0383)	(0.0505)
Constant	−4.772***	−5.676***
	(0.320)	(0.359)
Adjusted R2	0.403	0.0691
*N*	45,610	45,610
GP practice-fixed effects	Yes	Yes
Year fixed effects	Yes	Yes

Outcome variables and registered population size are inverse hyperbolic sine transformations of counts.

Robust standard errors in parentheses. *p < 0.10, **p < 0.05, ***p < 0.01

Amongst practices referring to the two Trusts which piloted the faster diagnosis standard for lung cancer, however, we estimate the pilot was associated with a statistically significant decrease in referral volumes for suspected lung cancer (0.105, *p* < 0.01). This corresponds to an average relative decrease in suspected lung cancer referral volumes of −10.5% for practices who referred all of their suspected cancer patients to a lung cancer pilot in the year before the pilot (i.e. treatment dose = 1), compared to practices who referred none of their suspected cancer patients to the two pilot Trusts.

#### Parallel trends and the implication of pre-trends for the estimated pilot effect

**Fig. 3:**
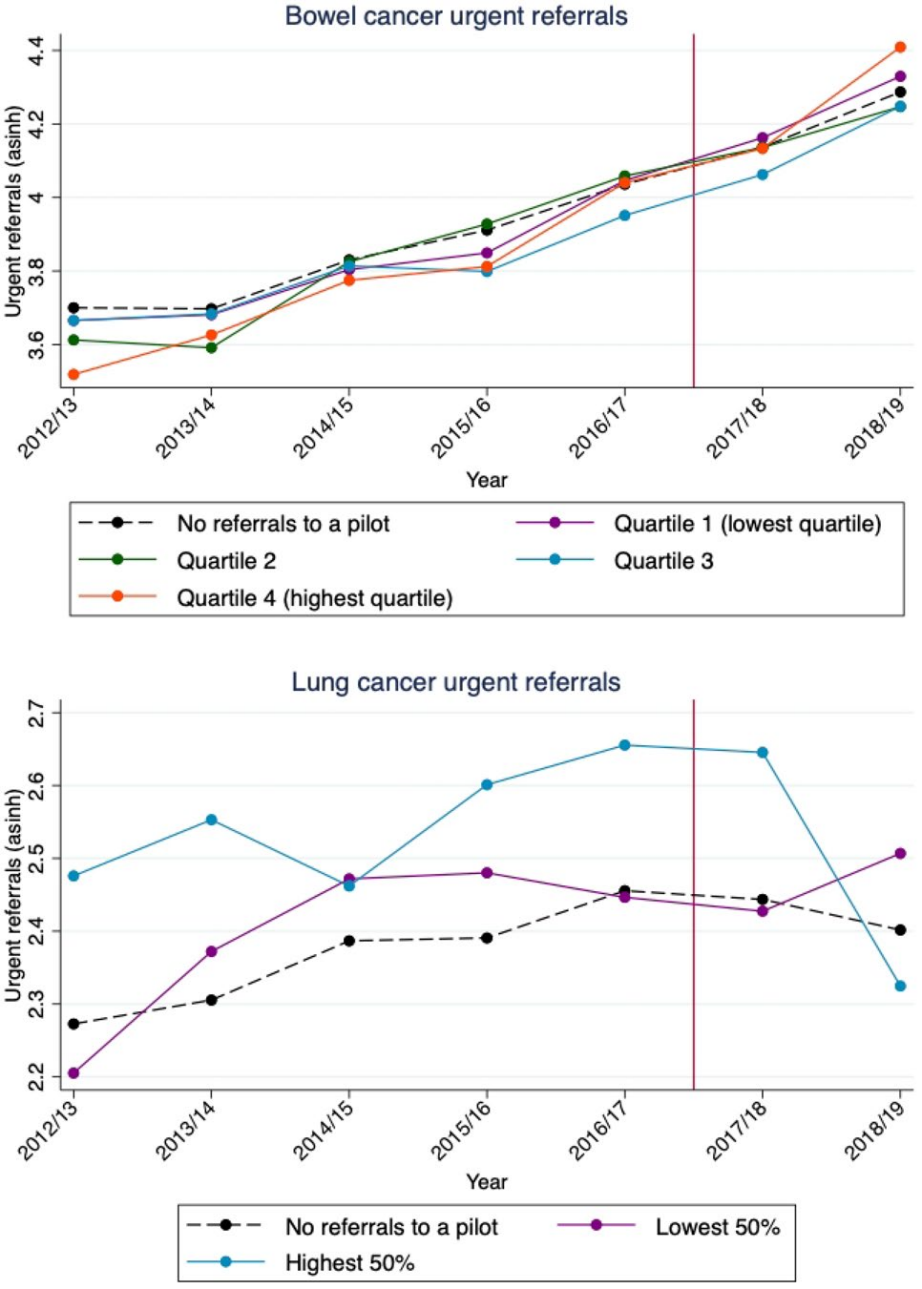
Adjusted plots of urgent referral volumes for bowel and lung cancer over time. Outcome variables are inverse hyperbolic sine transformations of counts. Graphs are adjusted for practice-level covariates. Only data with a positive value for the proportion of referrals going to a pilot are used to generate the quartiles. For the lower graph, practices are grouped into three categories due to lack of variation in the data. For bowel cancer, trends are presented in five groups (no referrals to a pilot in the year before the reform was introduced, and the remaining practices split into quartiles). For lung cancer, due to low variation in the data, trends are split into three groups (no referrals to a pilot, and the remaining practices split above and below the median)

For bowel cancer, GP referrals appear to be similar in levels across the groups of practices with different levels of exposure and there does not appear to be much divergence in trends before the pilot was introduced (Fig. 3). There is some visual evidence of an uptick in referrals in 2018/19 in the most exposed group (quartile 4). Table 7 shows the estimated divergence in trends in the pre-period is 0.018 (*p* > 0.1). This result is statistically insignificant, and small in magnitude in comparison with the pilot effect of 0.108 (*p* < 0.01). If we were to assume any divergence in pre-trends persists, then the true pilot effect may be slightly smaller than our estimated effect but would not remove the effect entirely.

**Table 7:** *F*-tests of pre-trends in GP referral volumes

	Bowel cancer	Lung cancer
Time trend*Treatment dose	0.0179	−0.00550
	(0.0140)	(0.0156)
Time trend	0.0879***	0.0457***
	(0.00196)	(0.00254)
Treatment dose	−36.20	11.27
	(28.23)	(31.46)
*N*	32,421	32,421

Null hypothesis: parallel trends in two-week wait referral volumes before the introduction of the pilot. Only observations in the pre-reform period (2012/13–2016/17) are included.

Robust standard errors in parentheses. *p < 0.10, **p < 0.05, ***p < 0.01

For lung cancer, we see an upward trend in referrals in the pre-period, and a large subsequent decrease in referral volumes in 2018/19 for practices with high exposure to a lung pilot hospital (Fig. 3). Table 7 shows the estimated divergence in pre-trends is -0.0055 (*p* > 0.1). This result is statistically insignificant, and small in magnitude in comparison with the pilot effect. Assuming this divergence in pre-trends persists into the post-period, then the estimated pilot effect (−0.105, *p* < 0.05) may overestimate the true effect very slightly. Again, however, the divergence in parallel trends would not be enough to explain the entire magnitude of the estimated effect.

### Robustness checks

Supplementary Table 3 shows the results of sensitivity analysis using breaches of the 62-day waiting times target as the outcome measure. We find consistent effects of the pilot in terms of direction and significance between the 62-day measure and the main waiting times analysis for bowel cancer, suggesting that waiting times for suspected bowel cancer did change as a result of the pilot. For lung cancer, we find a null effect of the pilot on both the 62-day and 14-day measures.

Supplementary Table 4 shows the results of our sensitivity analyses in relation to the impact of the pilot on waiting times. All analyses for bowel cancer support the main results in terms of the direction and significance of the effect, confirming a statistically significant decrease in waiting times. For lung cancer, all analyses confirm a statistically insignificant increase in waiting times, with the exception of the results of the waiting times analysis using the shorter pre-period, which supports the main results in terms of the direction of effect but with the magnitude now estimated to be larger and statistically significant. Supplementary Table 5 shows the pre-trends test is satisfied for the lung cancer waiting times analysis when we only include 3 years of data prior to the reform.

Supplementary Table 6 shows the results of our GP demand response. The direction of the detected effects is again consistent across all sensitivity analyses. The positive impact on bowel cancer referrals is found to be statistically significant across all sensitivity analyses, except where we examine only the first year of the pilot before Ipswich and Colchester Trusts merged. The negative impact on lung cancer referrals is found to be statistically significant across all sensitivity analyses, except the LDV model.

Sensitivity analysis examining changes in the definition of the treated group over time suggests there was not a statistically significant change in the proportion of practices’ referrals going to pilot hospitals in the year after the pilot was introduced (Supplementary Table 7). There are some changes to referral patterns in the years before our dose variable was defined. However, the magnitude of these changes is very small (between 0.5 and 2.6% of a standard deviation) and so the extent to which this introduces bias in our results is likely to be negligible.

## Discussion

### Summary of main findings

We examined the impact of a shorter waiting times pilot in England on the GP demand response for suspected cancer urgent referrals. We find that the pilot did reduce waiting times for bowel cancer and detect a corresponding increase in demand from GPs. These results provide evidence of a negative elasticity of GP demand with respect to waiting times for urgent cancer referral. Our results suggest that GPs respond to shorter waiting times by referring more marginal patients in the hope of ruling out cancer, and that this mechanism dominates any safety net responses where GPs may feel they have more scope to explore other alternatives before referral.

For lung cancer, the pilot was not found to have achieved its aim of reducing waiting times. We detect, if anything, an increase in the magnitude of waiting times breaches for patients with suspected lung cancer associated with the pilot, although this effect was not statistically significant in the majority of our analyses. We find evidence of a corresponding reduction in demand from GPs for suspected lung cancer referrals, which would be consistent with worsening waiting times. These results are also suggestive of a negative elasticity of GP demand with respect to waiting times. However, the results for lung cancer should be interpreted with caution given the lack of evidence of a significant change in waiting times amongst lung cancer pilot sites.

Characteristics of different cancer types may explain why the pilot succeeded in reducing waiting times for bowel, but not lung cancer. The slow-moving nature of some bowel cancers could explain why there is more capacity for waiting time improvements for bowel cancers, if suspected lung cancer patients were already prioritised prior to the pilot. The slow-moving nature of some bowel cancers could also explain why our findings fit with the literature on responses for non-urgent/elective surgery.

### Strengths and weaknesses of the study

This is the first study to examine the GP demand response to waiting times for a definitive cancer diagnosis. This study provides evidence on the debate surrounding early detection of cancer, and adds to the literature on waiting times by investigating demand responses in an urgent care setting. The increase in GP urgent referrals for suspected bowel cancer shows that policies which aim to reduce waiting times can have demand effects.

There is some evidence that the parallel trends assumption may not be met in some models. However, we do not find that the magnitude of the divergence of trends in the pre-period is enough to explain the entire magnitude of the estimated treatment effect in any case. Furthermore, sensitivity analyses using a LDV model support the findings of our main analysis for bowel cancer. For lung cancer waiting times, the sensitivity analysis using an LDV model also concurs with the results of our main analysis, again failing to detect a significant impact of the pilot on lung cancer waiting times. This suggests the lack of effect found in the main analysis is not due to violation of the parallel trends assumption.

The causal pathway we hypothesise is that the pilot affected waiting times, which in turn induced a GP demand response. However, the process may be dynamic. An initial drop in waiting times could generate a GP demand response, which would then feed back into increased waiting times. Using a relatively short window of time after the reform should help to reduce this bias, if we expect that the pilot affects GP demand for diagnostic services only through its effect on waiting times.

In examining changes to waiting times at the hospital level, we use waiting time breaches as a proxy for longer waiting times. However, if providers are stacking patients under the target [53], breaches could be falling but average waiting times could still rise. Data on average waiting times are not publicly available, but provider-level adherence to waiting times targets is regularly published. Furthermore, waiting time breaches are measured against operational standards which take into account things like patient delay or clinical appropriateness of treating within the target [54], which may not be possible to properly adjust for using average waiting times.

Data were not available on breaches of the 28-day target during our study period. However, the primary aim of the paper is to estimate the GP demand response to waiting times, and the waiting times analysis is used to check for a signal of whether waiting times were indeed affected by the pilot, in a way that was observable to GPs. Waiting time breaches of the 14-day standard were published by NHS England, whilst at the time, breaches to the 28-day standard were not. Waiting times for the first 14 days of the pathway were, therefore, more directly observable to GPs. Sensitivity analysis which looks at the effect of the pilot on breaches of the 62-day target (which includes the target 28 days between urgent referral and receipt of a diagnosis) supports the findings of our main waiting times analyses.

### Implications for policymakers and future research

Efforts to shorten waiting times for cancer diagnosis is a policy priority. Policymakers should be aware reforms may fail to achieve their aims in the long term, as the increase in capacity may eventually be offset by increased demand. Although even a temporary decrease in waiting times may represent a positive outcome, the NHS requires sustainable improvements to tackle poor survival rates in comparison to other high-income countries [55]. Given NHS England’s plans to simplify the number of cancer waiting time targets further [56], more robust evidence is needed on the implications of the removal of these incentives and changes to waiting time targets.

Missed cancer waiting times targets have greatly increased during the coronavirus pandemic [57]. Disruption of cancer services are predicted to continue for many years due to the patient backlog created by the sharp drop in diagnosis and treatment of cancer patients particularly during the first wave of the COVID-19 pandemic [58, 59]. As cancer services face increased strain in coming years, policymakers must consider the impact of increased waiting times on GP demand for these services in plans to address this backlog.

### Supplementary Information

Below is the link to the electronic supplementary material.

Supplementary file1 (DOCX 434 KB)

Supplementary file2 (DOCX 34 KB)

## Data Availability

The dataset for the Waiting Times analysis is available upon request from the corresponding author at hhayes@ohe.org. The dataset for the GP Referrals analysis cannot be shared publicly as data from Hospital Episode Statistics (HES) were used to link pilot hospital Trusts to GP practices, and a license is required for use of these data.

## References

[1] Cullis, J.G., Jones, P.R., Propper, C.: Chapter 23 Waiting lists and medical treatment Analysis and policies. Handb Health Econ. **1**, 1201–1249 (2000). Doi: 10.1016/S1574-0064(00)80036-0

[2] Siciliani L (2014). Waiting Times. Encyclopedia of Health Economics.

[3] Nikolova S, Harrison M, Sutton M (2016). The impact of waiting time on health gains from surgery: Evidence from a national patient-reported outcome dataset. Health Econ. UK..

[4] Martin S, Smith PC (1999). Rationing by waiting lists: An empirical investigation. J. Public Econ..

[5] Hauck K, Street A (2007). Do targets matter? A comparison of English and Welsh national health priorities. Health Econ..

[6] Riganti A, Siciliani L, Fiorio CV (2017). The effect of waiting times on demand and supply for elective surgery: Evidence from Italy. Health Econ. UK..

[7] Gravelle H, Dusheiko M, Sutton M (2002). The demand for elective surgery in a public system: Time and money prices in the UK National Health Service. J. Health Econ..

[8] Martin S, Rice N, Jacobs R, Smith P (2007). The market for elective surgery: Joint estimation of supply and demand. J. Health Econ..

[9] Siciliani L, Iversen T (2012). Waiting times and waiting lists. The elgar companion to health economics.

[10] Sripa P, Hayhoe B, Majeed A, Greenfield G, Garg P (2019). Impact of GP gatekeeping on quality of care, and health outcomes, use, and expenditure. Br. J. Gen. Pract..

[11] Foot, C., Naylor, C., Imison, C.: The quality of GP diagnosis and referral

[12] Raymont, A., Morgan, S., Mcleod, D., Dowell, A., Van Rij, A., Cumming, J., Pledger, M., Dew, K., Cormack, D.: The new zealand medical journal New Zealand general practitioners’ non-urgent referrals to surgeons: who and why? (2008) 18551154

[13] Kennedy F, McConnell B (1993). General practitioner referral patterns. J. Public Health..

[14] Windmeijer F, Gravelle H, Hoonhout P (2005). Waiting lists, waiting times and admissions: An empirical analysis at hospital and general practice level. Health Econ..

[15] Badrick E, Cresswell K, Ellis P, Renehan AG, Crosbie EJ (2019). Top ten research priorities for detecting cancer early. Lancet.

[16] Laudicella M, Walsh B, Burns E, Smith PC (2016). Cost of care for cancer patients in England: Evidence from population-based patient-level data. Br. J. Cancer..

[17] Harrison, C.J., Spencer, R.G., Shackley, D.C.: Transforming cancer outcomes in england: Earlier and faster diagnoses, pathways to success, and empowering alliances. J. Healthc. Leadersh. **11**, (2019). 10.2147/JHL.S150924 10.2147/JHL.S150924 PMC6357885 30774494

[18] Santos R, Gravelle H, Propper C (2017). Does quality affect patients’ choice of doctor?. Evid. Engl. Econ. J..

[19] Morris, J.: Cancer waiting times: How has NHS performance changed over time?, https://www.nuffieldtrust.org.uk/news-item/cancer-waiting-times-how-has-nhs-performance-changed-over-time

[20] Palmer, C.: Diagnosing cancer faster, https://www.england.nhs.uk/blog/focusing-on-cancer-wait-times/

[21] Cancer Research UK: Bowel cancer statistics, https://www.cancerresearchuk.org/health-professional/cancer-statistics/statistics-by-cancer-type/bowel-cancer

[22] Cancer Research UK: Lung cancer statistics, https://www.cancerresearchuk.org/health-professional/cancer-statistics/statistics-by-cancer-type/lung-cancer#heading-One

[23] Selvachandran S, Hodder R, Ballal M, Jones P, Cade D (2002). Prediction of colorectal cancer by a patient consultation questionnaire and scoring system: A prospective study. Lancet.

[24] NHS: Lung cancer, https://www.nhs.uk/conditions/lung-cancer/

[25] The American Cancer Society: Colon and Rectal Cancer Guide, https://www.cancer.org/cancer/colon-rectal-cancer/if-you-have-colon-rectal-cancer.html

[26] Parkin, E.: NHS maximum waiting time standards. (2021)

[27] NHS England: Statistics - Monthly provider based data and summaries, https://www.england.nhs.uk/statistics/statistical-work-areas/cancer-waiting-times/monthly-prov-cwt/

[28] Gruber J, Hoe TP, Stoye G (2023). Saving lives by tying hands: The unexpected effects of constraining health care providers. Rev. Econ. Stat..

[29] Smyth, C., Wright, O.: Tory party conference: NHS bosses face sack for failing to cut waits, https://www.thetimes.co.uk/article/tory-party-conference-nhs-bosses-face-sack-for-failing-to-cut-waits-gk26rgqm9, (2023)

[30] Allen T, Whittaker W, Sutton M, Kontopantelis E (2018). Influence of financial and reputational incentives on primary care performance: A longitudinal study. Br. J. Gen. Pract..

[31] Propper, C., Sutton, M., Whitnall, C., Windmeijer, F.: Did “Targets and Terror” reduce waiting times in England for hospital care? Cent. Mark. Public Organ. (2007)

[32] NHS Improvement: 28 Day faster diagnosis standard, https://improvement.nhs.uk/documents/1162/Session_2-B2_Mercian_2-The_28_day_faster_diagnostic_-_a_chance_to_learn_more__EOfuWGS.pptx, (2017)

[33] NHS England: 28 Day faster diagnosis standard, https://www.england.nhs.uk/statistics/2021/08/13/28-day-faster-diagnosis-standard/

[34] Miles A, Olsson L (2018). the psychological implications of diagnostic delay in colorectal cancer patients. Timely diagnosis of colorectal cancer.

[35] Quality Health: National Cancer Patient Experience Survey (2016)

[36] Smith, E.: Our work to help GPs spot the potential warning signs of cancer, https://scienceblog.cancerresearchuk.org/2014/02/10/our-work-to-help-gps-spot-the-potential-warning-signs-of-cancer/

[37] Campbell, D.: GP practices “offered rewards” for not referring patients to hospitals, https://www.theguardian.com/society/2015/oct/01/gp-practices-offered-rewards-for-not-referring-patients-to-hospitals

[38] Kaffash, J.: GPs offered up to 50% cut of savings generated by slashing their own referrals, https://www.pulsetoday.co.uk/news/referrals/gps-offered-up-to-50-cut-of-savings-generated-by-slashing-their-own-referrals/, (2018)

[39] Round T, Ashworth M, L’Esperance V, Møller H (2021). Cancer detection via primary care urgent referral and association with practice characteristics: a retrospective cross-sectional study in England from 2009/2010 to 2018/2019. Br. J. Gen. Pract..

[40] NHS England: Statistics - Quarterly Provider Based Cancer Waiting Times Statistics, https://www.england.nhs.uk/statistics/statistical-work-areas/cancer-waiting-times/quarterly-prov-cwt/

[41] NHS Digital: NHS Workforce Statistics, https://digital.nhs.uk/data-and-information/publications/statistical/nhs-workforce-statistics

[42] NHS England: Statistics - Bed Availability and Occupancy, https://www.england.nhs.uk/statistics/statistical-work-areas/bed-availability-and-occupancy/

[43] NHS Digital: Hospital Admitted Patient Care Activity, https://digital.nhs.uk/data-and-information/publications/statistical/hospital-admitted-patient-care-activity

[44] Roth, J.: Pre-test with Caution: Event-study estimates after testing for parallel trends I am grateful to. (2019)

[45] Rambachan, A., Roth, J.: A More Credible Approach to Parallel Trends*. (2022)

[46] Public Health England: Public Health Profiles, https://fingertips.phe.org.uk/

[47] NHS Digital: Patients Registered at a GP Practice, https://digital.nhs.uk/data-and-information/publications/statistical/patients-registered-at-a-gp-practice

[48] NHS England: GP Patient Survey, https://www.gp-patient.co.uk/

[49] Bellemare MF, Wichman CJ (2020). Elasticities and the inverse hyperbolic sine transformation. Oxf. Bull. Econ. Stat..

[50] NHS Digital: QOF 2019–20, https://qof.digital.nhs.uk/

[51] Card D (1992). Using regional variation in wages to measure the effects of the federal minimum wage. ILR Rev..

[52] O’Neill S, Kreif N, Grieve R, Sutton M, Sekhon JS (2016). Estimating causal effects: considering three alternatives to difference-in-differences estimation. Health Serv. Outcomes Res. Methodol..

[53] Bevan G, Hood C (2006). What’s measured is what matters: Targets and gaming in the English public health care system. Public Adm..

[54] Hamilton, M., Hodgson, O., Dai, D., Mcdonnell, P.: Waiting Times for Suspected and Diagnosed Cancer Patients 2017–18 Annual Report. NHS England (2018)

[55] Arnold C, Theede J, Gagnon A (2014). A qualitative exploration of access to urban migrant healthcare in Nairobi. Kenya. Soc. Sci. Med..

[56] Nuffield Trust: Cancer waiting times, https://www.nuffieldtrust.org.uk/resource/cancer-waiting-time-targets

[57] The Nuffield Trust: Cancer waiting times. (2021)

[58] Patel, P., Thomas, C.: Building back cancer services in England. Institute for Public Policy Research (2021)

[59] Davies, J.: What impact has Covid-19 had on cancer services?, https://www.nuffieldtrust.org.uk/news-item/what-impact-has-covid-19-had-on-cancer-services

