# Supplementary material for: How do family doctors respond to reduced waiting times for cancer diagnosis in secondary care?
Source: Eur J Health Econ. 2023 Oct 3;25(5):813–28. doi: 10.1007/s10198-023-01626-2 (PMC11192671; doi:10.1007/s10198-023-01626-2)
Supplement: Supplementary file 1 — Supplementary file1 (DOCX 434 KB) [file 10198_2023_1626_MOESM1_ESM.docx]

**Supplementary Figure 1: Histograms of the outcome variables before and after inverse hyperbolic sine transformations**

**
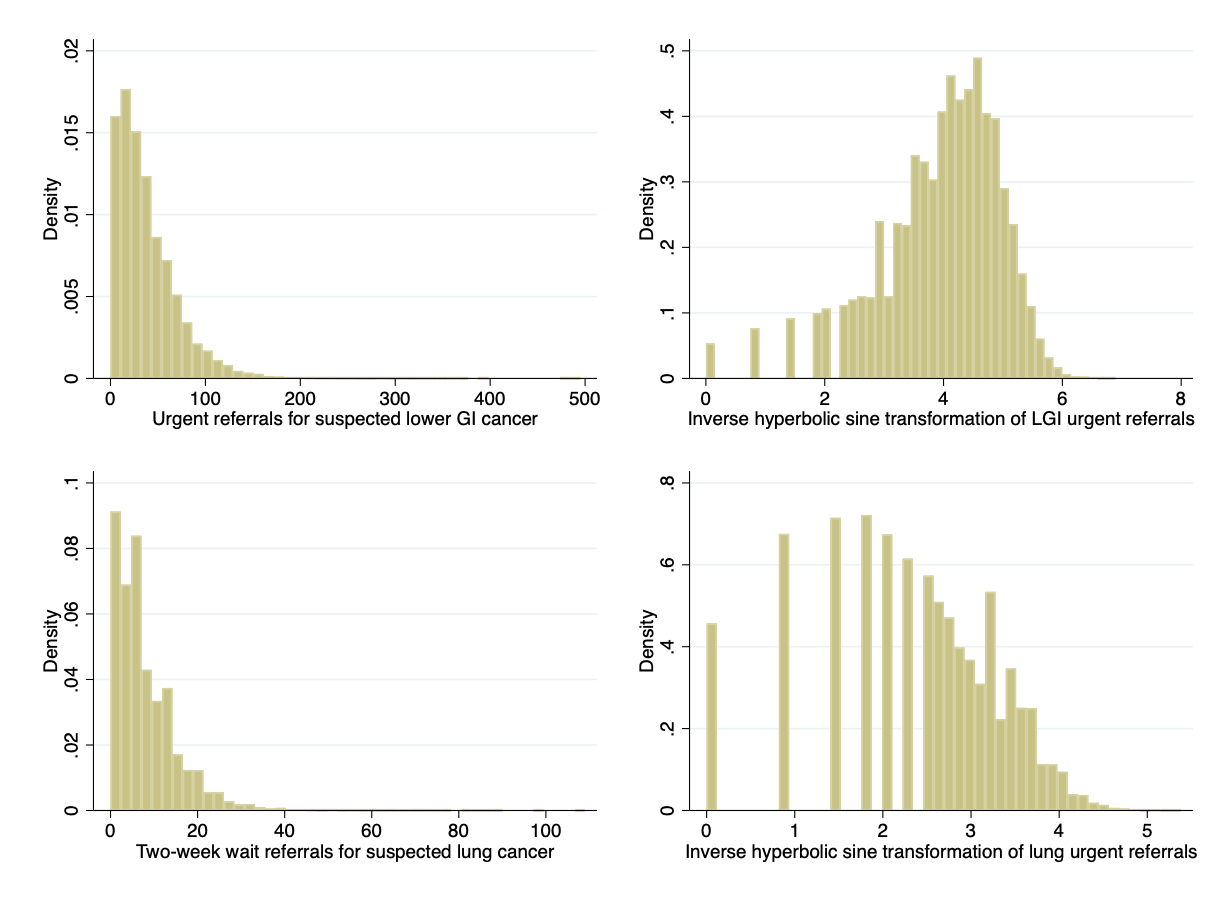
**

**Supplementary Figure 2: Share of general practice level referral appointments made to the first and second highest hospital provider of each general practice**

**
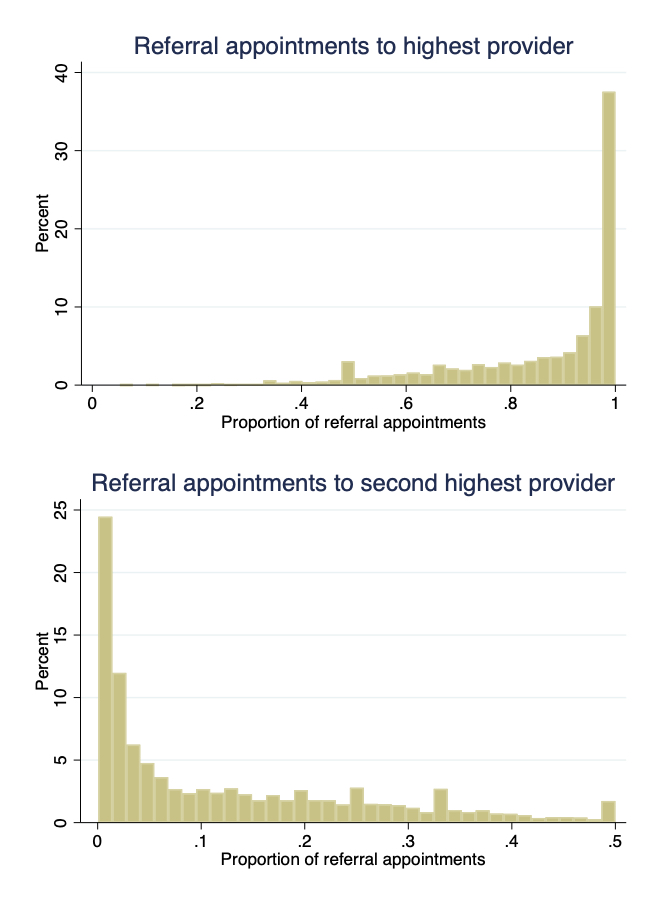
**
