# Supplementary material for: How do family doctors respond to reduced waiting times for cancer diagnosis in secondary care?
Source: Eur J Health Econ. 2023 Oct 3;25(5):813–28. doi: 10.1007/s10198-023-01626-2 (PMC11192671; doi:10.1007/s10198-023-01626-2)
Supplement: Supplementary file 2 — Supplementary file2 (DOCX 34 KB) [file 10198_2023_1626_MOESM2_ESM.docx]

**Supplementary Table 1: Cancer types for which the 28 day faster diagnosis standard was piloted in each pilot site**

| Cancer type | East Lancashire | Ipswich | Kingston | Leeds | Royal Bournemouth and Christchurch |
| --- | --- | --- | --- | --- | --- |
| Lung | X |  |  |  | X |
| Upper GI | X |  |  |  |  |
| Gynaecology |  | X | X | X |  |
| Bowel |  | X | X |  | X |
| Head and Neck |  |  |  | X |  |
| Urology |  |  |  | X | X |

(Palmer, 2017)

**Supplementary Table 2: Definitions and sources of variables used in the analysis**

| **Variable** | **Source** | **Definition** |
| --- | --- | --- |
| **Waiting times dataset** |  |  |
| *Outcomes* |  |  |
| Bowel breaches as a % of total suspected bowel cancer patients seen | Monthly provider based waiting time statistics, NHS England | The number of breaches of the 2WW rule for suspected bowel cancer as a % of the number seen by a specialist after urgent referral from GP for suspected bowel cancer |
| Lung breaches as a % of total suspected lung cancer patients seen | Monthly provider based waiting time statistics, NHS England | The number of breaches of the 2WW rule for suspected bowel cancer as a % of the number seen by a specialist after urgent referral from GP for suspected lung cancer |
| *Covariates* |  |  |
| Total beds | Bed availability and occupancy, NHS England | Average daily number of total beds available open overnight |
| Proportion beds occupied | Bed availability and occupancy, NHS England | Average daily number of total beds occupied as a proportion of total beds available (open overnight) |
| FTE staff |  |  |
| Number on waiting list | Hospital Episode Statistics, NHS Digital | The count of elective admission episodes indicating that the admission was from a waiting list, excluding planned admissions |
| Mean length of stay | Hospital Episode Statistics, NHS Digital | The average spell of continuous admitted patient care in days. A spell is defined by subtracting the admission date from the discharge date. |
|  |  |  |
| **GP response dataset** |  |  |
| *Outcomes* |  |  |
| Bowel urgent referrals | NHS England Cancer Waiting Times Database | The number of 2WW referrals for suspected bowel cancer made for the registered practice population |
| Lung urgent referrals | NHS England Cancer Waiting Times Database | The number of 2WW referrals for suspected lung cancer made for the registered practice population |
| *Covariates* |  |  |
| Registered population size | QOF, NHS Digital | QOF practice list size |
| Proportion aged 65+ years | Patients registered at a GP practice by NHS Digital. | Proportion of the registered practice population aged 65 years or over |
| Proportion aged under 18 years | Patients registered at a GP practice by NHS Digital. | Proportion of the registered practice population aged under 18 |
| Total QOF points achieved (proportion) | QOF, NHS Digital | QOF points achieved across all QOF domains as a proportion of all achievable points |
| Working status - Unemployed | GP Patient Survey | Q57: “Which of these best describes what you are doing at present” Proportion who answered “unemployed” |
| Proportion reporting good overall experience of making appointment | GP Patient Survey | Q22: "Overall, how would you describe your experience of making an appointment?". Proportion who answered either "Very good" or "Fairly good" |
| Proportion with a long-standing health condition | GP Patient Survey | Question 34. "Do you have any long-term physical or mental health conditions, disabilities or illnesses". Proportion who answered “yes”. |
| Proportion satisfied with phone access | GP Patient Survey | Question 1. People were asked: "Generally, how easy is it to get through to someone at your GP practice on the phone?". Proportion who answered "Very easy" or "Fairly easy" excluding those who answered "Haven't tried". |

Notes: QOF = Quality Outcomes Framework. 2WW= Two-Week Wait. All data for the GP response analysis were sourced from Public Profiles by Public Health England, the original sources for these data are referenced in the table and definitions as provided by Public Profiles.

**Supplementary table 3: Sensitivity analyses on the measure of waiting times**

|  | **Main waiting times analysis (breaches of the 14-day target)** | | 62-day breaches | |
| --- | --- | --- | --- | --- |
|  | **Bowel** | **Lung** | Bowel | Lung |
|  |  |  |  |  |
| Pilot effect | **-3.907***** | **1.695** | -7.112*** | -2.913 |
|  | **(1.344)** | **(1.31)** | (1.564) | (3.750) |
|  |  |  |  |  |
| Adjusted R2 | **0.0557** | **0.0339** | 0.131 | 0.175 |
| N | **825** | **955** | 825 | 954 |
| Provider fixed effects | **YES** | **YES** | YES | YES |
| Year fixed effects | **YES** | **YES** | YES | YES |

**Supplementary Table 4: Waiting times analysis: Results of sensitivity analyses**

|  |  |  | |  |  | | |
| --- | --- | --- | --- | --- | --- | --- | --- |
| Outcome | **Primary analysis** | Excluding covariates | LDV | Removing the first two years of data | Including anticipation year dummy (2016/17) | Excluding Ipswich and Colchester and including 2018/19 | Remove other pilots from the treatment group |
| Bowel cancer main pilot effect | **-3.907***** | -3.719*** | -4.396** |  | -4.188*** | -5.897*** | -3.920*** |
|  | **(1.344)** | (1.378) | (1.785) |  | (1.050) | (1.409) | (1.345) |
| Bowel cancer anticipation effect |  |  |  |  | -1.276 |  |  |
|  |  |  |  |  | (1.770) |  |  |
| Observations | **825** | 825 | 130 |  | 825 | 943 | 813 |
| Lung cancer main pilot effect | **1.695** | 1.769 | 1.338 | 3.238*** | 1.311 | - | 1.676 |
|  | **(1.310)** | (1.239) | (0.929) | (0.869) | (1.572) | - | (1.318) |
| Lung cancer anticipation effect |  |  |  |  | -1.905 |  |  |
|  |  |  |  |  | (1.333) |  |  |
| Observations | **956** | 955 | 256 | 671 | 955 | - | 936 |
| Provider fixed effects | **YES** | YES | NO | YES | YES | YES | YES |
| Year fixed effects | **YES** | YES | YES | YES | YES | YES | YES |
| Lags | **NO** | NO | YES | NO | NO | NO | NO |
| Covariates | **YES** | NO | YES | YES | YES | YES | YES |

Notes: The first row for bowel cancer and lung cancer includes the coefficient of interest for each model (the pilot effect) and robust standard error in parentheses below. The anticipation year model includes the 2016/17 year dummy pilot effect below the main pilot effect. All models including covariates control for FTE all staff, total beds, the proportion of occupied beds, waiting list size and mean length of stay. For the LDV model the outcomes are waiting time breaches as a % of total suspected cancer appointments.

**Supplementary Table 5: F-tests of parallel trends for the lung cancer DiD waiting times analysis, removing the first two years of data**

|  | **Lung cancer** |
| --- | --- |
|  |  |
| Time trend*Treatment dummy | 0.273 |
|  | (0.552) |
|  |  |
| Time trend | 0.0147 |
|  | (0.182) |
|  |  |
| Treatment dummy | -552.8 |
|  | (1111.1) |
|  |  |
| N | 408 |

**Supplementary Table 6: GP demand response: Results of sensitivity analyses**

| Outcome | **Primary analysis** | LDV | Including anticipation year dummy (2016/17) | Including Ipswich and Colchester and excluding 2018/19 | Excluding Ipswich and Colchester and Including 2018/19 | Removing other pilot locations from the control group |
| --- | --- | --- | --- | --- | --- | --- |
| Bowel cancer urgent referrals | **0.108***** | 0.0357* | 0.124*** | 0.0475 | 0.118** | 0.0989*** |
|  | **(0.0358)** | (0.0202) | (0.0405) | (0.0395) | (0.0486) | (0.0355) |
|  |  |  | 0.0816** |  |  |  |
|  |  |  | (0.0333) |  |  |  |
| Observations | **45610** | 12878 | 45610 | 39017 | 44633 | 44681 |
| Lung cancer urgent referrals | **-0.105**** | -0.0529 | -0.111** | - | - | -0.107** |
|  | **(0.0460)** | (0.0403) | (0.0501) | - | - | (0.0461) |
|  |  |  | -0.0256 |  |  |  |
|  |  |  | (0.0498) |  |  |  |
| Observations | **45610** | 12878 | 45610 | - | - | 43431 |
| Practice fixed effects | **YES** | NO | YES | YES | YES | YES |
| Year fixed effects | **YES** | YES | YES | YES | YES | YES |
| Lags | **NO** | YES | NO | NO | NO | NO |

Notes: The first row for bowel cancer and lung cancer include the coefficient of interest for each model (the pilot effect) and robust standard error in parentheses below. The anticipation year model includes the 2016/17 year dummy pilot effect below the main pilot effect. All models control for practice list size, proportion age 65+, proportion aged under 18, proportion with a long-standing health condition, proportion unemployed, proportion reporting good experience of making an appointment, proportion satisfied with phone access and QOF points. Outcomes and practice list size are inverse hyperbolic sine transformations of counts.

* p<0.10, ** p<0.05, *** p<0.01

**Supplementary Table 7: Test for changes in practice referral patterns to pilot hospital Trusts 2012/13-2017/18**

|  | **Bowel cancer** | **Lung cancer** |
| --- | --- | --- |
|  |  |  |
| 2012/13 | 0.000830 | 0.00327*** |
|  | (0.000797) | (0.000799) |
|  |  |  |
| 2013/14 | 0.00122 | 0.00284*** |
|  | (0.000780) | (0.000780) |
|  |  |  |
| 2014/15 | -0.00117*** | 0.000321 |
|  | (0.000384) | (0.000477) |
|  |  |  |
| 2015/16 | 0.000678* | -0.000470 |
|  | (0.000378) | (0.000327) |
|  |  |  |
| 2016/17 | 0 | 0 |
|  | (.) | (.) |
|  |  |  |
| 2017/18 | 0.000301 | -0.000470 |
|  | (0.000306) | (0.000392) |
|  |  |  |
| Adjusted R2 | 0.000609 | 0.00244 |
| N | 39017 | 39017 |
| GP practice fixed effects | YES | YES |
| Covariates | YES | YES |

Notes: 2016/17 is the base category. All models control for practice list size, proportion age 65+, proportion aged under 18, proportion with a long-standing health condition, proportion unemployed, proportion reporting good experience of making an appointment, proportion satisfied with phone access and QOF points.
